# Competition for recruitment in SARS-CoV-2 Trials in the United States: a longitudinal cohort analysis

**DOI:** 10.1186/s13104-022-06263-1

**Published:** 2022-12-12

**Authors:** Nora Hutchinson, Katarzyna Klas, Benjamin G. Carlisle, Maciej Polak, Jonathan Kimmelman, Marcin Waligora

**Affiliations:** 1 Studies of Translation, Ethics, and Medicine (STREAM), Biomedical Ethics Unit, McGill University, Montreal, QC Canada; 2 Research Ethics in Medicine Study Group (REMEDY), Faculty of Health Sciences, Jagiellonian University Medical College, Michalowskiego 12, PL-31-126 Krakow, Poland; 3 Berlin Institute of Health at Charité (BIH), BIH QUEST Center for Transforming Biomedical Research, Berlin, Germany; 4 Department of Epidemiology and Population Studies, Institute of Public Health, Faculty of Health Sciences, Jagiellonian University Medical College, Kraków, Poland

**Keywords:** Clinical trials, Patient-participant recruitment, Competition, SARS-CoV-2, COVID-19, United States

## Abstract

**Objective:**

Competition among trials for patient enrollment can impede recruitment. We hypothesized that this occurred early in the COVID-19 pandemic, when an unprecedented number of clinical trials were launched. We performed a simple and multivariable regression analysis evaluating the relationship between the proportion of SARS-CoV-2 investigational trial sites within each USA state with unsuccessful patient-participant recruitment and: (i) the proportion of cases required to reach state recruitment goals; (ii) state population based on data from the US Census; and, (iii) number of trial sites per state.

**Results:**

Our study included 151 clinical trials. The proportion of trials with successful recruitment was 72.19% (109 of 151 trials). We did not find a significant relationship between unsuccessful patient-participant recruitment, state recruitment goals, state population or the number of trial sites per state in both our simple and multivariable regression analyses. Our results do not suggest that early in the COVID-19 pandemic, competition for patient-participants impeded successful recruitment in SARS-CoV-2 trials. This may reflect the unique circumstances of the first few months of the pandemic in the United States, in which the number and location of SARS-CoV-2 cases was sufficient to meet trial recruitment requirements, despite the large number of trials launched.

## Introduction

In the first year of the pandemic the international research community launched an unprecedented number of clinical trials directed at the treatment and prevention of SARS-CoV-2 [1, 2]. Despite the large volume of documented SARS-CoV-2 cases, we hypothesized that competition for trial participants may have negatively impacted patient-participant recruitment.

Concerns regarding inadequate patient-participant recruitment leading to early trial termination predate the COVID-19 pandemic [3–7]. Recent studies have evaluated the proportion of SARS-CoV-2 trials reaching 75% of goal recruitment [8] and have compared trial recruitment targets and feasible recruitment numbers in England [9]. One study evaluated geographic alignment of SARS-CoV-2 cases and interventional clinical trial sites in the United States, noting decreasing alignment between trials sites and new cases as the pandemic progressed [10].

In what follows we evaluate patient-participant recruitment of SARS-CoV-2 treatment trials registered on ClinicalTrials.gov, with a site in the United States, and assess dependency of unsuccessful trial recruitment and state recruitment goals. We consider whether local competition between trials may have impacted recruitment success.

## Main text

### Methods

#### Trial sample

We included interventional SARS-CoV-2 treatment efficacy trials registered on ClinicalTrials.gov with a trial site in the United States (USA) and a start date between 2020–01-01 and 2020–06-30. We excluded trials evaluating interventions aimed at the prevention of SARS-CoV-2. Prevention trials typically enroll healthy volunteers, which was not the population of interest in our study. We included all eligible trials in our cohort; therefore, no sample size calculation was performed. Data were downloaded from the web front-end of ClinicalTrials.gov on 2020–12-01 and again on 2021–01-04, allowing us to evaluate patient-participant recruitment results at the 6-month mark from date of trial start (see [11] for further elaboration of our methods).

#### Data curation

We evaluated patient-participant recruitment for each trial in our cohort. Pre-specified criteria for unsuccessful recruitment were: (i) trial was “terminated” or “suspended” for a reason unrelated to efficacy, safety or progression of science (an automated extraction of the “why_stopped” element from ClinicalTrials.gov trial registration records was performed, enabling this categorization); (ii) trial was “completed” or “active, not recruiting” with a final enrollment less than 85% of the anticipated enrollment cited in the registration record at trial start, thus reflecting a substantial loss of statistical power for the primary outcome [4]; or, (iii) trial was “recruiting” or “enrolling by invitation” and the recruitment period had been extended to at least twice as long as anticipated, based on the planned recruitment length at trial start.

We aggregated trial recruitment goals on a state-by-state basis, and then compared this to the number of contemporaneous active SARS-CoV-2 cases in each state between 2020–01-01 and 2020–06-30. This involved (i) estimating the goal patient-participant enrollment per state for each trial (dividing planned enrollment per trial by the total number of sites per trial and multiplying this by the number of trial sites per USA state); (ii) aggregating total patient-participant enrollment goals per state; (iii) estimating the number of active SARS-CoV-2 cases per state between 2020–01-01 and 2020–06-30 using data downloaded from usafacts.org; and, (iv) calculating the proportion of SARS-CoV-2 cases that would need to be recruited to fulfill aggregate trial enrollment goals by state. Multisite trials with at least 1 site in the USA were included in our analysis. Patient-participant enrollment numbers were prorated up to 2020–06-30 for trials not achieving primary outcome completion by this date.

#### Outcomes and statistical analysis

We report the proportion of trials with successful patient-participant recruitment, as well as the proportion of SARS-CoV-2 cases required to fulfill trial recruitment goals by state. For all states with two or more active trials during our 6-month time period, we performed a simple and multivariable regression analysis evaluating the relationship between the proportion of trial sites within each USA state with unsuccessful patient-participant recruitment and: (i) the proportion of cases required to reach state recruitment goals; (ii) state population based on data from the US Census [12]; and, (iii) number of trial sites per state. We hypothesized that increased state recruitment goals would result in an increase in unsuccessful patient-participant recruitment. To investigate this further, a robust regression was performed using the M-estimation with Huber weights [13]. This specific type of regression analysis was chosen due to the continuous nature of the dependent variable and our detection of influential observations based on the Cook distance. The results of regression analysis were presented as beta coefficient (b) with 95% confidence interval (95% CI). Robust regressions were performed using the robustbase package [14], R version 4.1.0 (2021) [15]. We defined *p* < 0.05 as statistically significant.

## Results

We included 151 interventional SARS-CoV-2 treatment trials in our cohort. The majority (68.21%) were Phase 2; 129 (85.43%) were randomized. Study status at 6 months since trial start was “Recruiting” in 99 trials (65.56%) (Table 1). Median anticipated enrollment per trial was 152 patients (IQR 60—400). The proportion of trials with successful patient-participant recruitment was 72.19% (109 of 151 trials).

**Table 1:** Characteristics of trial cohort

Category	Number of trials (N = 151)	Percent total (%)	Median (IQR) anticipated enrollment^a^	Median (IQR) actual enrollment^b^
Trial phase				
Phase 2^c^	103	68.21	100 (44–200)	48 (20–100)
Phase 3^d^	48	31.79	400 (263–600)	243 (143–1088)
Randomization				
Randomized	129	85.43	200 (60–400)	110 (41–236)
Non-randomized	8	5.30	68 (20–158)	20 (18–23)
NA^e^	14	9.27	47 (26–100)	33 (13–49)
Trial status^f^				
Completed	12	7.95	397 (58–503)	81 (29–1075)
Terminated	4	2.65	279 (54–625)	51 (36–63)
Active, not recruiting	27	17.88	120 (43–289)	86 (32–175)
Recruiting	99	65.56	138 (60–329)	196 (20–225)
Enrolling by invitation	4	2.65	400 (230–515)	None
Suspended	5	3.31	500 (200–600)	1 (1–1)
Sponsorship				
Industry sponsor	51	33.77	200 (84–385)	130 (59–231)
Non-industry sponsor	100	66.23	136 (50–500)	50 (19–225)
Number of centers				
Single center	58	38.41	100 (41–255)	24 (17–50)
Multicenter	93	61.59	208 (80–400)	140 (50–243)

a) Anticipated enrollment in the first registration record after trial start

b) At the 6-month mark, for the subset of trials which provide actual enrollment information

c) Includes Phase 1/2

d) Includes Phase 2/3

e) NA—Information not available in the ClinicalTrials.gov registration record

f) Trial Status at the 6-month mark since trial start

Forty-seven of 50 states launched at least one SARS-CoV-2 treatment trial. Three states (Kansas, Vermont and North Dakota) only had 1 trial location per state and were excluded from our linear analysis. California had the greatest number of trial sites per state (268), followed by New York (204) and Texas (197). Fourteen states and the District of Columbia required an enrollment of at least 1% of all persons with SARS-CoV-2 in their region to fulfill recruitment goals (Table 2). The elevated New Mexico recruitment requirement was mostly driven by a single-site clinical trial (NCT04458948) with an anticipated enrollment of 10,000 participants.

**Table 2:** Percent recruitment required per state

Location	Target enrollment	SARS-CoV-2 Cases	Percent recruitment required (%)
New Mexico	2734	12147	22.51
Hawaii	76	917	8.25
Utah	688	22372	3.07
Montana	24	985	2.46
Minnesota	709	36299	1.95
West Virginia	54	2885	1.89
District of Columbia	162	10327	1.57
Maine	50	3292	1.52
California	3984	299438	1.33
Washington	430	32822	1.31
New York	4799	393496	1.22
Missouri	283	25483	1.11
Oregon	93	8656	1.07
Michigan	758	70725	1.07
North Carolina	677	64668	1.05

Data are presented for the 14 states and the District of Columbia that had recruitment targets ≥ 1% of total SARS-CoV-2 cases

We did not find any significant relationship between unsuccessful patient-participant recruitment, increased state recruitment goals, state population or the number of trial sites per state in our simple and multivariable regression analyses (Table 3).

**Table 3:** Simple and multivariable robust regression analyses

	Univariable analysis			Multivariable analysis		
	b	95% CI	P-value	b	95% CI	P-value
State recruitment goals	− 0.23	− 0.70–0.25	0.30	-0.27	− 0.87–0.34	0.38
State population (per 1 M)	2.0 × 10^− 5^	− 3.5 × 10^− 3^–3.5 × 10^–3^	0.99	1.5 × 10^− 3^	− 8.7 × 10^− 3^–0.01	0.77
Number of trials	− 1.0 × 10^− 5^	− 5.0 × 10^− 4^–4.0 × 10^− 4^	0.96	− 2.0 × 10^− 4^	− 1.5 × 10^− 3^–1.1 × 10^− 3^	0.73

Relationship between unsuccessful patient-participant recruitment, state recruitment goals, state population and the number of trial sites per state

*b* beta coefficient from robust regression, *CI* confidence interval

## Discussion

Most trials in our cohort demonstrated successful patient-participant recruitment (72.19%; 109 of 151 trials). Fourteen states and the District of Columbia required the enrollment of at least 1% of the SARS-CoV-2 cases in their jurisdiction to fulfill recruitment goals. Prior research in oncology has demonstrated that trials with a lower enrollment fraction (percentage of eligible patient-participants needed to fulfill goal trial enrollment) are more likely to successfully accrue [3]. However, the level of competition for participants in USA oncology trials appears to be greater than was seen early in the pandemic. Across all cancer types, approximately 8% of newly diagnosed patients in the USA would need to enroll in an interventional oncology trial to fulfill trial recruitment goals [16]. In comparison, the percentage recruitment requirements per state in our study were modest (Table 2), which is in keeping with our regression analyses that did not demonstrate a significant correlation between state-level recruitment targets and unsuccessful trial recruitment.

Our results differ from those of Franks et al. who noted declining alignment between SARS-CoV-2 cases and trial sites during the course of the pandemic, as evidenced by an increase in the number of counties with significant case numbers without local trial sites, as well as an upward trend in counties with a high volume of trial sites for the number of local cases [10]. These findings were most pronounced after June 2020. Our study focused on the first 6 months of the pandemic, which may explain why we did not find a significant relationship between recruitment goals and unsuccessful patient-participant recruitment.

## Conclusion

Others have criticized a lack of coordination in SARS-CoV-2 trial activation early in the pandemic [2, 17, 18]. Despite these early failures in trial coordination, our findings did not demonstrate a significant relationship between competition for a limited pool of participants and under-accrued clinical trials. This may reflect the unique circumstances of the first few months of the pandemic in the United States, in which the number and location of SARS-CoV-2 cases was sufficient to meet trial recruitment requirements.

## Limitations

Our study has four main limitations. First, we assumed absence of interstate and international travel in our calculation of the proportion of patient-participants required to fulfill trial enrollment goals by state. Given reduced travel during the early months of the pandemic due to stay-at-home orders adopted in the majority of USA states and territories, [19] this seemed a reasonable assumption. Second, we estimated competition for patient-participant trial enrollment at a state level, rather than at a county level, under the assumption that a degree of inter-county travel was maintained in the setting of trial SARS-CoV-2 clinical care and trial recruitment. Third, for multi-site trials we assumed equal goal patient-participant enrollment per trial site, due to lack of public access to site-specific enrollment goals. Fourth, we relied on data from ClinicalTrials.gov and usafacts.org to estimate trial feasibility and to model state level trial recruitment dynamics. Our results are thus dependent on the accuracy of both ClincalTrials.gov registration records and usafacts.org 2020 SARS-CoV-2 daily new case counts.

## Data Availability

The data sets used in this analysis can be freely and openly accessed on Open Science Framework under 10.17605/OSF.IO/FP726. Please see reference [20] for link to the data.
